# Compositional change of gut microbiome and osteocalcin expressing endothelial progenitor cells in patients with coronary artery disease

**DOI:** 10.1371/journal.pone.0249187

**Published:** 2021-03-25

**Authors:** Takumi Toya, Ilke Ozcan, Michel T. Corban, Jaskanwal D. Sara, Eric V. Marietta, Ali Ahmad, Irina E. Horwath, Darrell L. Loeffler, Joseph A. Murray, Lilach O. Lerman, Amir Lerman

**Affiliations:** 1 Department of Cardiovascular Medicine, Mayo Clinic, Rochester, MN, United States of America; 2 Division of Cardiology, National Defense Medical College, Tokorozawa, Saitama, Japan; 3 Division of Gastroenterology and Hepatology, Mayo Clinic, Rochester, MN, United States of America; 4 Division of Nephrology and Hypertension, Mayo Clinic, Rochester, MN, United States of America; Medical University Innsbruck, AUSTRIA

## Abstract

Osteogenic endothelial progenitor cells (EPCs) contribute to impaired endothelial repair and promote coronary artery disease (CAD) and vascular calcification. Immature EPCs expressing osteocalcin (OCN) has been linked to unstable CAD; however, phenotypic regulation of OCN-expressing EPCs is not understood. We hypothesized that gut-microbiome derived pro-inflammatory substance, trimethylamine N-oxide (TMAO) might be associated with mobilization of OCN-expressing EPCs. This study aimed to investigate the association between dysbiosis, TMAO, and circulating mature and immature OCN-expressing EPCs levels in patients with and without CAD. We included 202 patients (CAD N = 88; no CAD N = 114) who underwent assessment of EPCs using flow cytometry and gut microbiome composition. Mature and immature EPCs co-staining for OCN were identified using cell surface markers as CD34+/CD133-/kinase insert domain receptor (KDR)+ and CD34-/CD133+/KDR+ cells, respectively. The number of observed operational taxonomy units (OTU), index of microbial richness, was used to identify patients with dysbiosis. The number of immature OCN-expressing EPCs were higher in patients with CAD or dysbiosis than patients without. TMAO levels were not associated with circulating levels of OCN-expressing EPCs. The relative abundance of *Ruminococcus gnavus* was moderately correlated with circulating levels of immature OCN-expressing EPCs, especially in diabetic patients. Gut dysbiosis was associated with increased levels of TMAO, immature OCN-expressing EPCs, and CAD. The relative abundance of *Ruminococcus gnavus* was correlated with immature OCN-expressing EPCs, suggesting that the harmful effects of immature OCN-expressing EPCs on CAD and potentially vascular calcification might be mediated by gut microbiome-derived systemic inflammation.

## Introduction

The endothelium is a primary site for the deleterious effects of cardiovascular risk factors [1]. Damaged endothelium can be repaired by peripherally mobilized endothelial progenitor cells (EPCs) from bone marrow to maintain endothelial homeostasis. Thus, quantification of circulating EPCs levels may reflect the vascular reparative capacity [2]. The imbalance between vascular damage and repair leads to endothelial dysfunction, an early manifestation of atherosclerosis [1,3]. Not only decreased circulating levels but also dysfunction of EPCs may promote endothelial dysregulation. We previously reported that retention of osteoblastic marker, osteocalcin (OCN), expressing EPCs in the coronary circulation was associated with coronary endothelial dysfunction [4]. CD34+ cells from subjects with high OCN expression were able to form mineralized deposits with calcium, suggesting their involvement in vascular calcification [5]. In addition, increased levels of circulating OCN-expressing EPCs were associated with coronary artery disease (CAD) and calcified aortic stenosis [5–8]. Interestingly, an immature subset of OCN-expressing EPCs, defined as CD34-/CD133+/KDR+ cells, was more strongly associated with unstable CAD than a mature subset, defined as CD34+/CD133-/KDR+ cells, indicating its involvement in abnormal vascular repair [6]. However, the phenotypic regulation of OCN-expressing EPCs is not understood.

Growing evidence revealed that the symbiotic relationship between gut microbiome and its host shapes our health through multiple mechanisms; thus dysbiosis of gut microbiome has been implicated to a variety of diseases and conditions such as inflammatory/immune diseases, metabolic diseases, and malignancies [9–12]. Consistent with previous studies, we have also reported the close association between compositional change of the gut microbiome, such as an increase in the relative abundance of *Ruminococcus gnavus*, and CAD [13,14]. *Ruminococcus gnavus* has also been linked to diabetes mellitus and inflammatory diseases (inflammatory bowel disease, spondyloarthritis, and eczema) [15–19]. Another possible link between microbiome and CAD could be mediated through a pathway including trimethylamine-N-oxide (TMAO), which is generated by oxidization of gut microbiome-produced trimethylamine from carbon sources such as lecithin, choline, betaine, and carnitine [20]. Elevated plasma levels of TMAO were associated with a higher risk of major adverse cardiovascular events (MACE) potentially through phenotypic modulation of M1/M2 macrophage, inducing endothelial dysfunction and platelet activation [21–24]. A recent study showed that TMAO levels were negatively correlated with circulating plasma EPC numbers and endothelial function evaluated by flow-mediated dilatation, and positively correlated with inflammatory markers such as high-sensitivity C-reactive protein and interleukin-1β in patients with stable angina. Higher TMAO levels were associated with lower MACE-free survival, whereas plasma EPC numbers did not predict MACE in that study [25]. The same authors also reported that incubation of cultured EPCs with TMAO promoted cellular inflammation, elevated oxidative stress, and suppressed tube formation and migration of EPCs in vitro, indicating that phenotypic alteration of EPCs may provide prognostic value beyond the amount of EPCs [25].

We hypothesized that gut microbiome-derived substances might be linked to dysregulation of EPCs with resultant increase in CAD. This study aimed to investigate the association between dysbiosis, TMAO, and circulating levels of OCN-expressing EPCs with specific focus on mature and immature subsets of OCN-expressing EPCs.

## Materials and methods

### Study population

We enrolled 202 consecutive patients who underwent assessment of gut microbiome composition and EPCs between December 2013 and November 2018. Of 202 patients, 133 were referred to Mayo Clinic for clinically indicated coronary angiography for the assessment of chest pain and/or dyspnea. The remaining 69 patients were patients who had abnormal peripheral endothelial dysfunction assessed by reactive hyperemia peripheral arterial tonometry (RH-PAT) without known or suspected CAD based on clinical history, non-invasive stress testing, and coronary computed tomography and/or coronary angiography. Subjects with prior gastrointestinal surgery including colectomy, ileectomy, and gastrectomy, the current administration of antibiotics or probiotics, or a known history of inflammatory bowel disease or auto-immune diseases, were not eligible for the study. We applied the results of the previous paper, demonstrating that 2–4 μmol/L increase in TMAO was associated with endothelial dysfunction and EPCs dysregulation with a resultant increase in MACE, to calculate the sample size using PS: Power and Sample Size Calculation Program Version 3.1.6 [25]. We anticipated that the samples of 31 cases in CAD group would be required to test our hypothesis at the probability of 80% and a two-sided 0.05 significance level. Thus, we ultimately underwent the current analyses with 114 non-CAD patient and 88 CAD patients. The study was conducted in accordance with the guidelines of the Declaration of Helsinki and was approved by the Mayo Clinic Institutional Review Board. All participants provided written informed consent.

### Clinical assessment

Clinical history, laboratory data, and current medications list were collected from detailed chart reviews by an investigator blinded to the microbiome and EPC data. Data were collected on the following parameters: 1) sex, age, body mass index, smoking status, and obesity, 2) dyslipidemia, defined by a documented history of hyperlipidemia, treatment with lipid-lowering therapy, a low-density lipoprotein cholesterol above target (<130 mg/dL for low risk patients, <100 mg/dL for moderate-high risk patients, <70 mg/dL for very high risk, and <55 mg/dL for extreme high risk patients based on 10-year atherosclerotic cardiovascular disease risk), high-density lipoprotein cholesterol <40 mg/dL in men or <50 mg/dL in women, or triglyceride >150 mg/dL, 3) type 2 diabetes mellitus, defined as a documented history or treatment of type 2 diabetes, 4) hypertension, defined as a documented history of or treatment for hypertension, and 5) CAD, defined as a documented history of percutaneous coronary intervention or coronary artery bypass grafting for significant coronary artery stenosis, or more than 50% of luminal stenosis in any coronary arteries diagnosed by coronary angiography or computed tomography coronary angiography [14].

### Assessment of peripheral endothelial function

The peripheral endothelial function was evaluated by RH-PAT, as previously described [26,27]. Briefly, the study protocol included a 5-minute baseline measurement, followed by 5-minute inflation of a blood pressure cuff around the test arm with a pressure of 60 mm Hg above baseline systolic blood pressure up to 200 mm Hg, followed by a 6-minute PAT measurement after deflation of the cuff. Blood pressure cuff occlusion was not applied to the control arm (contralateral arm). RH-PAT ratio was determined as the average pulse wave amplitude for a 1-minute-period beginning 1 minute after pressure cuff deflation divided by the average pulse wave amplitude during a 3.5-minute baseline period. The RH-PAT index was calculated automatically through a computer algorithm by normalizing baseline signal and indexing the RH-PAT ratio on the test arm to that of the control arm. A calculated RH- PAT index ≤2.0 was used as a cut-off value for the diagnosis of peripheral endothelial dysfunction [26,27].

### Assessment of gut microbiome composition

16S rDNA of collected stool samples were analyzed to investigate the compositional change of microbiome using the method described previously [14,28]. In brief, participants were provided with stool collection kits (Fisher Scientific Inc., Pittsburgh, PA, USA). If patients were unable to give the stool samples during their stay at Mayo Clinic, they were instructed to collect the sample at home and ship it through overnight mail delivery. Samples were frozen at -70°C within 24 hours of receipt. DNA extraction from stool, amplicon (V3-V5 region) generation, and sequencing was done by the University of Minnesota Genomics Center. 16S sequencing data was run through the IM-TORNADO pipeline to form operational taxonomic units (OTU) at a 97% similarity level [29]. Alpha-diversity measures were calculated based on the rarefied OTU counts. The number of observed OTU and Chao-1 index indicates microbial richness, which measures the number of taxa in each sample. Shannon index indicates microbial evenness, which measures the relative number of taxa in samples accounting for the number of times each taxon was observed in a sample. Given that there were no predefined cut-off values of alpha-diversity measures to diagnose dysbiosis, dysbiosis or symbiosis were defined as observed OTU <median or ≥median in this study.

### Assessment of circulating endothelial progenitor cells

Flow cytometry was performed to assess circulating endothelial progenitor cell counts as previously described [4,5,30]. In brief, peripheral blood mononuclear cells were isolated from fresh blood samples obtained in EDTA tubes using a Ficoll density gradient. Immunofluorescent cell staining was undertaken with the following fluorescent conjugated antibodies: CD34-PerCP Cy 5.5 (Becton Dickinson), KDR allophycocyanin (R&D Systems), CD133/2-phycoerythrin (Miltenyi Biotec GmbH), and the appropriate isotype controls. Osteocalcin (OCN)-expressing cells were recognized using an anti-human OCN antibody (Santa Cruz Biotechnology) with a fluorescein isothiocyanate secondary antibody (Jackson ImmunoResearch) as previously described elsewhere [5]. Live cells were detected using propidium iodide exclusion (Becton Dickinson). Cell fluorescence was measured after staining (FACSCalibur; Becton Dickinson). Data were analyzed using CellQuest software (Becton Dickinson). We aimed to capture 150,000 events, and final data were acquired in the lymphocyte gate with examples as previously described [4,7]. Results were expressed as counts per 100,000 events and log transformed (LN). Circulating cells expressing CD34 and KDR but not CD133 were considered mature EPCs (CD34+/CD133-/KDR+ cells), and cells expressing CD133 and KDR but not CD34 were classified as immature EPCs (CD34-/CD133+/KDR+ cells) [6].

### TMAO measurement

TMAO concentrations were measured in plasma samples obtained on the same day of EPC assessment and determined by liquid chromatography-mass spectrometry, as previously described [31,32]. Briefly, a solution of D9-isotopes was spiked in plasma samples as an internal standard. The mixture was deproteinized with cold methanol. The supernatant was dried down and resuspended in running buffer prior to injecting on a Sciex 6500 triple quadrupole mass spectrometer (Framingham, MA) coupled with a Cohesive TX2 LC system (Franklin, MA). Analytes were separated on a Grace Altima HP HILIC 150mm x 2.1 mm, 5 mm column prior to analyzing on the mass spectrometer via positive electrospray ionization mode. The analytes were separated on the analytical column using a gradient buffer system with buffer A being 99% water, 1% methanol and 10 mM Ammonium formate; and B being 100% methanol, 0.5% formic acid, and 1 mM Ammonium formate. Data acquisition was made using selective ion monitoring. The concentration of each analyte was calculated against an 8-point standard curve for each perspective analyte.

### Statistical analysis

Continuous variables distributed normally were expressed as the mean ± standard deviation, and those with a skewed distribution were expressed as the median with interquartile range. Categorical variables were expressed as frequency (percentage). For between-groups comparisons, unpaired t-test for normally distributed continuous variables, a Mann-Whitney U test (or Kruskal-Wallis test with post hoc test using Dunn method) for non-normally distributed variables, and χ^2^ test (or Fisher exact test) for categorical variables. Correlation between two normally-distributed variables was assessed using Pearson’s correlation test. Relative abundance of the microbe was log transformed to follow normal distribution. A 2-tailed *P* value <0.05 was considered statistically significant. All statistical analyses were performed using JMP Pro software 14.3.0 (SAS Institute, Inc., Cary, NC, USA) and GraphPad Prism 8.3.0 (GraphPad Software, La Jolla, California, USA).

## Results

### Baseline characteristics

**Fig 1:**
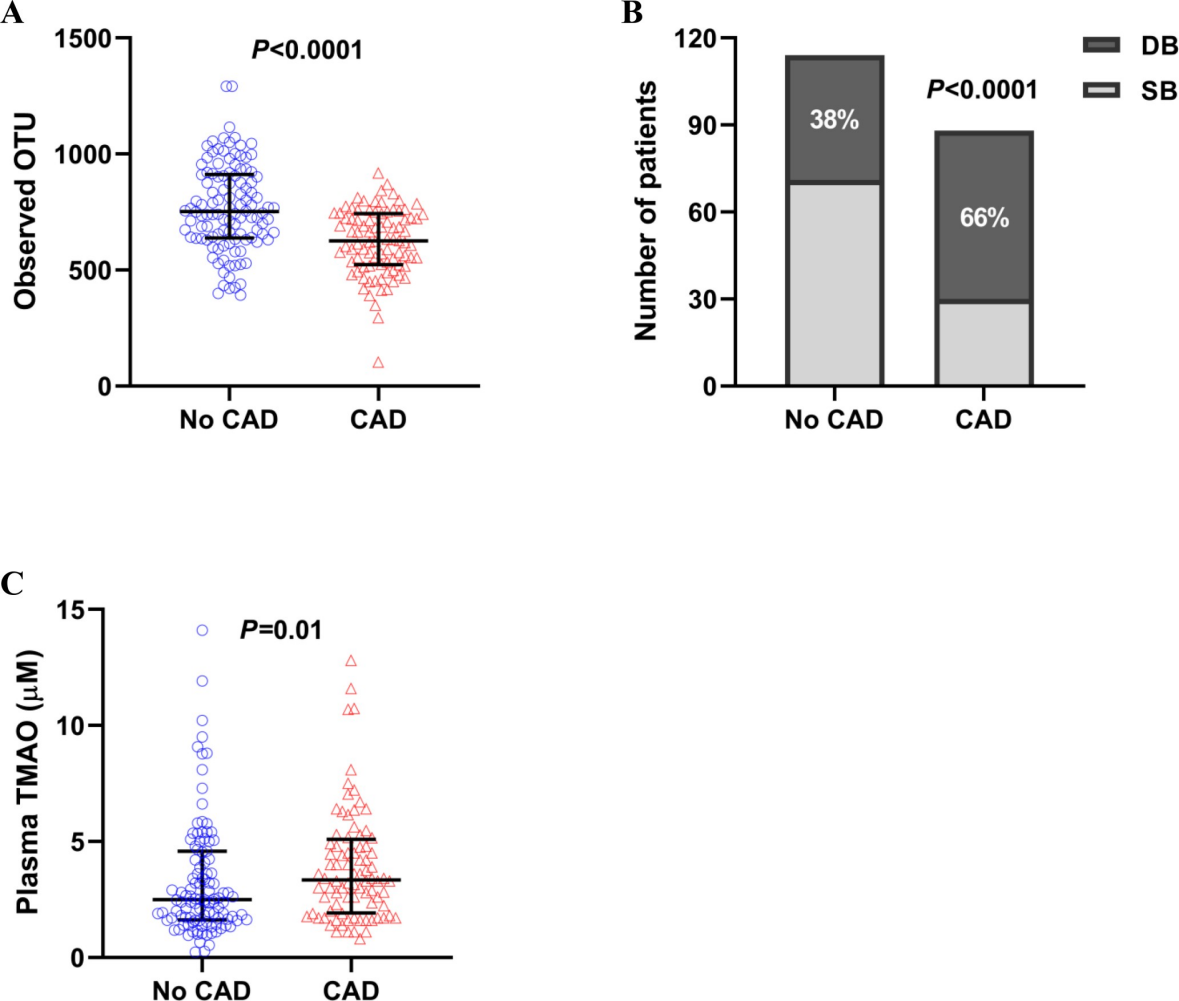
Comparison of gut microbiome composition, TMAO levels between patients with and without CAD. (A) Observed OTU was significantly lower in patients with CAD (N = 88) than those without CAD (N = 114) (Mann-Whitney U test *P*<0.0001). (B) The prevalence of dysbiosis was significantly higher in patients with CAD than those without CAD (58/88 [66%] vs. 43/114 [38%], χ^2^ test *P*<0.0001). (C) Plasma levels of TMAO were significantly higher in patients with CAD (N = 88) than those without CAD (N = 112) (Mann-Whitney U test *P* = 0.01). CAD, coronary artery disease; DB, dysbiosis; OTU, operational taxonomy unit; SB, symbiosis; TMAO, trimethylamine N-oxide.

**Fig 2:**
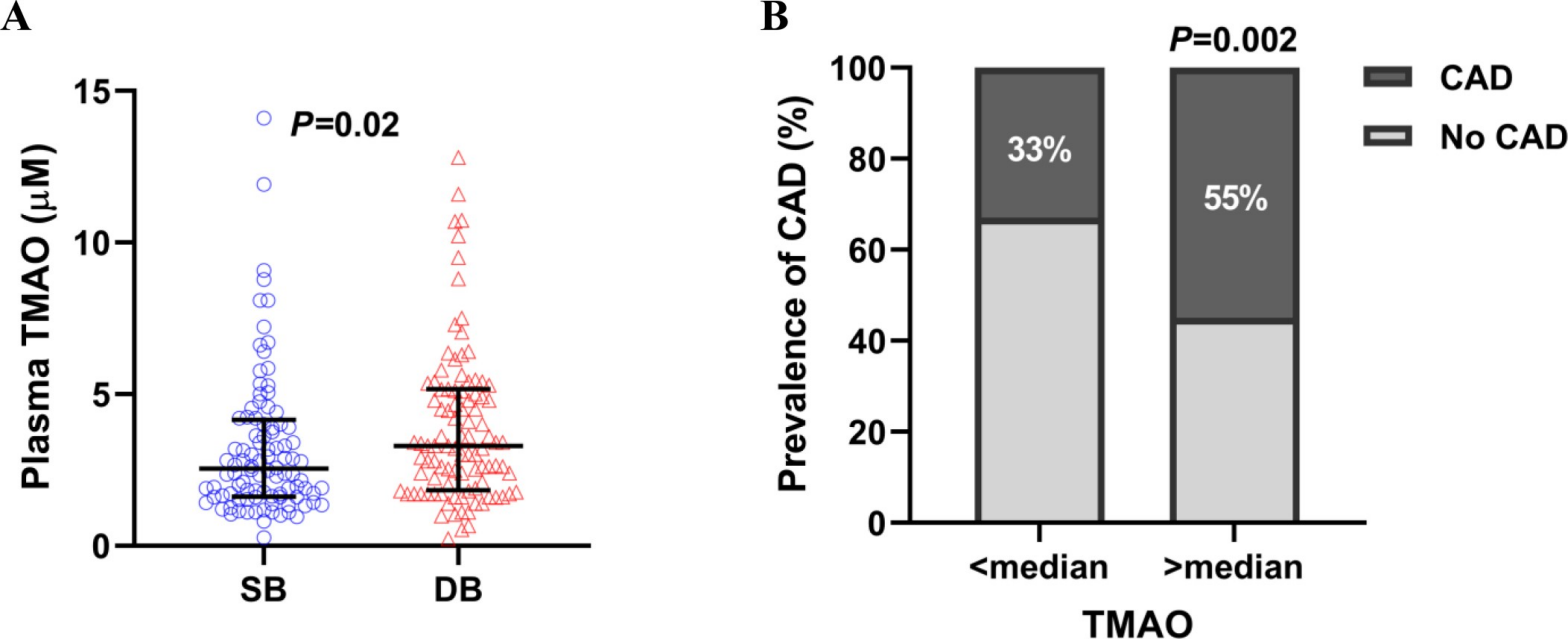
Association between gut microbiome composition, TMAO levels, and CAD. (A) TMAO levels were higher in patients with dysbiosis (N = 100) than those without (N = 100) (Mann-Whitney U test *P* = 0.02). (B) CAD was more prevalent in patients with >median levels of TMAO than those with <median levels of TMAO (55/100 (55%) vs. 33/100 (33%), χ^2^ test P = 0.002). CAD, coronary artery disease; DB, dysbiosis; TMAO, trimethylamine N-oxide.

Baseline characteristics are summarized in **Table 1**. The mean age was 55.7±16.1 years, 113 patients (56%) were males, and 197 (98%) were white. Patients with CAD (88 patients, 44%) and dysbiosis (101 patients, 50%) were significantly older, more likely to be males and obese, and had conventional cardiovascular risk factors (hypertension, dyslipidemia, and diabetes mellitus) more than those without CAD or dysbiosis. Observed OTU was significantly lower in patients with than those without CAD (626 [523, 743] vs. 752 [639, 912], *P*<0.0001) (**Fig 1A** and **Table 1**). Dysbiosis was more prevalent in patients with CAD than those without (N = 58 [66%] vs. N = 43 [38%], *P*<0.0001) (**Fig 1B**) in line with higher plasma levels of TMAO (available in 88 patients with CAD and 112 patients without CAD) in patients with CAD than those without (3.4 [1.9, 5.1] μM vs. 2.5 [1.6, 4.6] μM, *P* = 0.01) (**Fig 1C**). Similarly, patients with dysbiosis had higher levels of TMAO than those without (3.3 [1.8, 5.2] μM vs. 2.5 [1.6, 4.2] μM, *P* = 0.02) (**Fig 2A**). Detailed comparison of gut microbiome composition between patients with and without CAD was shown in our previous report [14].

**Table 1:** Baseline characteristics comparing patients with and without CAD.

	Total N = 202	No CAD N = 114	CAD N = 88	*P* value	No dysbiosis N = 101	Dysbiosis N = 101	*P* value
Age, years	55.7±16.1	48.2±16.3	65.4±9.3	<0.0001	51.0±17.7	60.4±12.9	<0.0001
Male sex, N (%)	113 (56)	46 (40)	67 (76)	<0.0001	48 (48)	65 (64)	0.02
White race, N (%)	197 (98)	109 (96)	88 (100)	0.05	97 (96)	100 (99)	0.17
Comorbidities, N (%)							
Hypertension	78 (39)	29 (25)	49 (56)	<0.0001	28 (28)	50 (50)	0.002
Diabetes Mellitus	25 (12)	2 (2)	23 (26)	<0.0001	5 (5)	20 (20)	0.001
Dyslipidemia	88 (44)	26 (23)	62 (70)	<0.0001	32 (32)	56 (56)	0.001
Smoking, N (%)							
Current	112 (55)	75 (66)	37 (42)	0.003	61 (60)	51 (50)	0.06
Former	76 (38)	32 (28)	44 (50)		37 (37)	39 (39)	
Never	14 (7)	7 (6)	7 (8)		3 (3)	11 (11)	
Laboratory data							
LDL-C, mg/dL	98 (76, 129)	103 (80, 136)	86 (70, 104)	0.002	99 (76, 130)	95 (77, 123)	0.58
HDL-C, mg/dL	53 (43, 67)	58 (49, 75)	43 (36, 53)	<0.0001	55 (46, 70)	50 (42, 67)	0.21
Tryglyceride, mg/dL	100 (74, 142)	92 (66, 128)	117 (92, 178)	0.0002	95 (64, 136)	112 (84, 170)	0.01
FPG, mg/dL	95 (88, 107)	90 (84, 97)	110 (102, 142)	<0.0001	90 (84, 102)	103 (92, 126)	<0.0001
Creatinine, mg/dL	0.96±0.22	0.92±0.19	1.01±0.23	0.003	0.93±0.22	0.98±0.22	0.12
BMI, kg/m^2^	29.1±6.1	27.4±5.8	31.2±5.9	<0.0001	26.9±5.4	31.2±6.1	<0.0001
Chao-1	896 (744, 1020)	939 (818, 1116)	804 (676, 948)	<0.0001	1020 (947, 1143)	744 (640, 821)	<0.0001
Shannon index	6.1 (5.6, 6.4)	6.2 (5.8, 6.6)	5.9 (5.4, 6.3)	<0.0001	6.4 (6.2, 6.6)	5.6 (5.2, 5.9)	<0.0001
Observed OTU	710 (579, 802)	752 (639, 912)	626 (523, 743)	<0.0001	802 (750, 922)	579 (496, 641)	

BMI, body mass index; CAD, coronary artery disease; HDL-C, high-density lipoprotein cholesterol; LDL-C, low-density lipoprotein cholesterol; FPG, fasting plasma glucose; OTU, operational taxonomy units.

### CAD and OCN-expressing EPCs

**Fig 3:**
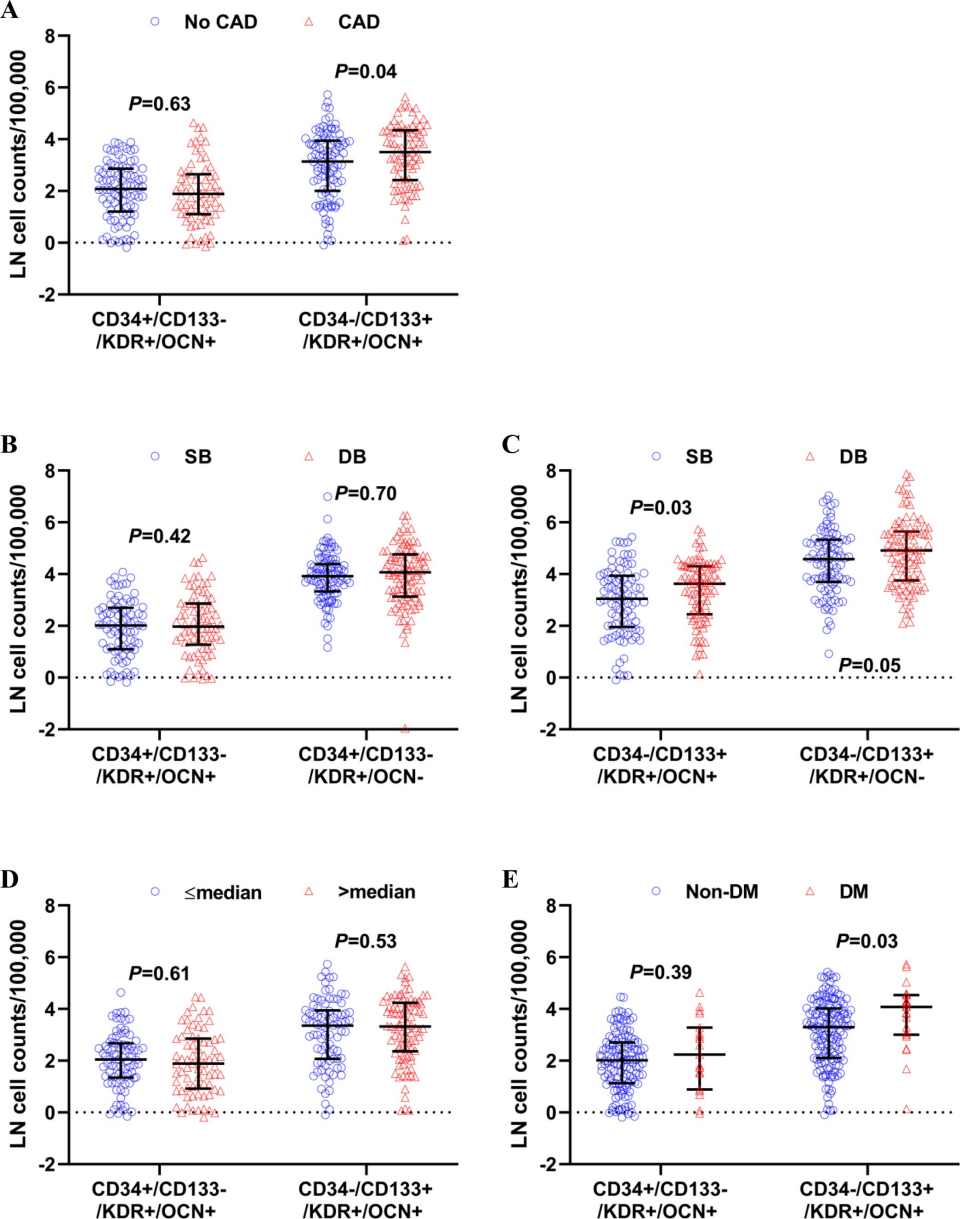
Association between CAD, dysbiosis, TMAO, and OCN-expressing EPCs. (A) Circulating CD34+/CD133-/KDR+/OCN+ cell levels were not different between patients with (N = 88) and without CAD (N = 114) (unpaired t-test *P* = 0.63), whereas circulating CD34-/CD133+/KDR+/OCN+ cell levels were higher in patients with CAD (N = 88) than those without CAD (N = 114) (unpaired t-test *P* = 0.04). (B) Circulating levels of CD34+/CD133-/KDR+/OCN+ and CD34+/CD133-/KDR+/OCN- cells were not different between patients with (N = 101) and without dysbiosis (N = 101) (unpaired t-test *P* = 0.42 and *P* = 0.70, respectively). whereas circulating CD34-/CD133+/KDR+/OCN+ cell levels were higher in patients with dysbiosis (N = 101) than those without dysbiosis (N = 101) (unpaired t-test *P* = 0.03). (C) Circulating CD34-/CD133+/KDR+/OCN+ cell levels were significantly higher in patients with dysbiosis than those without (*P* = 0.03). Circulating CD34-/CD133+/KDR+/OCN- cell levels tended to be higher in patients with dysbiosis than those without (*P* = 0.05). (D) Circulating levels of CD34+/CD133-/KDR+/OCN+ cells and CD34-/CD133-/KDR+/OCN+ cells were not different between patients with >median and <median levels of TMAO (*P* = 0.61 and *P* = 0.53, respectively). (E) Circulating CD34+/CD133-/KDR+/OCN+ cell levels were not different between patients with and without DM (*P* = 0.39), whereas circulating CD34-/CD133+/KDR+/OCN+ cell levels were higher in patients with DM than those without DM (*P* = 0.03). CAD, coronary artery disease; DB, dysbiosis; DM, diabetes mellitus; EPC, endothelial progenitor cell; OCN, osteocalcin; SB, symbiosis.

First, we investigated the association between CAD and OCN-expressing EPCs. Levels of circulating “mature OCN-expressing EPCs” (CD34+/CD133-/KDR+/OCN+ cells) were not different between patients with and without CAD (1.89 [1.10, 2.65] vs. 2.08 [1.20, 2.86] LN cell counts/100,000 counts, *P* = 0.63) (**Fig 3A**). In contrast, circulating “immature OCN-expressing EPC” (CD34-/CD133+/KDR+/OCN+ cell) levels were significantly higher in patients with CAD than those without CAD (3.50 [2.42, 4.35] vs. 3.14 [1.00, 3.95] LN cell counts/100,000 counts, *P* = 0.04) (**Fig 3A**).

### Dysbiosis and OCN-expressing EPCs

Next, we investigated the association between dysbiosis and OCN-expressing EPCs. Levels of circulating CD34+/CD133-/KDR+/OCN+ cells were not different between patients with and without dysbiosis (1.97 [1.27, 2.87] vs. 2.01 [1.10, 2.70] LN cell counts/100,000 counts, *P* = 0.42) (**Fig 3B**). In contrast, circulating CD34-/CD133+/KDR+/OCN+ cell levels were significantly higher in patients with than those without dysbiosis (3.63 [2.45, 4.30] vs. 3.04 [1.95, 3.94] LN cell counts/100,000 counts, *P* = 0.03) (**Fig 3C**). Levels of immature EPCs without expression of OCN also tended to be higher in patients with than those without dysbiosis (4.91 [3.75, 5.64] vs. 4.58 [3.70, 5.33] LN cell counts/100,000 counts, *P* = 0.05), while levels of mature EPCs without expression of OCN were not different between them (4.07 [3.13, 4.77] vs. 3.92 [3.33, 4.39] LN cell counts/100,000 counts, *P* = 0.70) (**Fig 3B and 3C**). Comparison of mature and immature OCN-expressing EPC levels in CAD vs non-CAD patients with or without dysbiosis revealed that immature OCN-expressing EPC levels were higher in CAD patients with dysbiosis than those in non-CAD patients without dysbiosis (*P* = 0.01), while there was no significant difference in mature OCN-expressing EPC levels among 4 groups (*P* = 0.55) (**S1** and **S2 Figs**).

### TMAO and OCN-expressing EPCs

Patients with >median levels of TMAO (N = 100) had a higher prevalence of CAD than those with <median levels of TMAO (N = 100) (N = 55 (55%) vs. N = 33 (33%), *P* = 0.002) (**Fig 2B**). We hypothesized that TMAO might be the link between the dysbiosis and OCN-expressing EPCs. However, levels of circulating CD34+/CD133-/KDR+/OCN+ cells and CD34-/CD133-/KDR+/OCN+ cells were not different between patients with >median and <median levels of TMAO (LN CD34+/CD133-/KDR+/OCN+ cell counts/100,000 counts, 1.89 [0.92, 2.85] vs. 2.05 [1.35, 2.67], *P* = 0.61; LN CD34-/CD133+/KDR+/OCN+ cell counts/100,000 counts, 3.32 [2.36, 4.23] vs. 3.35 [2.07, 3.94], *P* = 0.53) (**Fig 3D**).

### *Ruminococcus gnavus* and immature OCN-expressing EPCs

**Fig 4:**
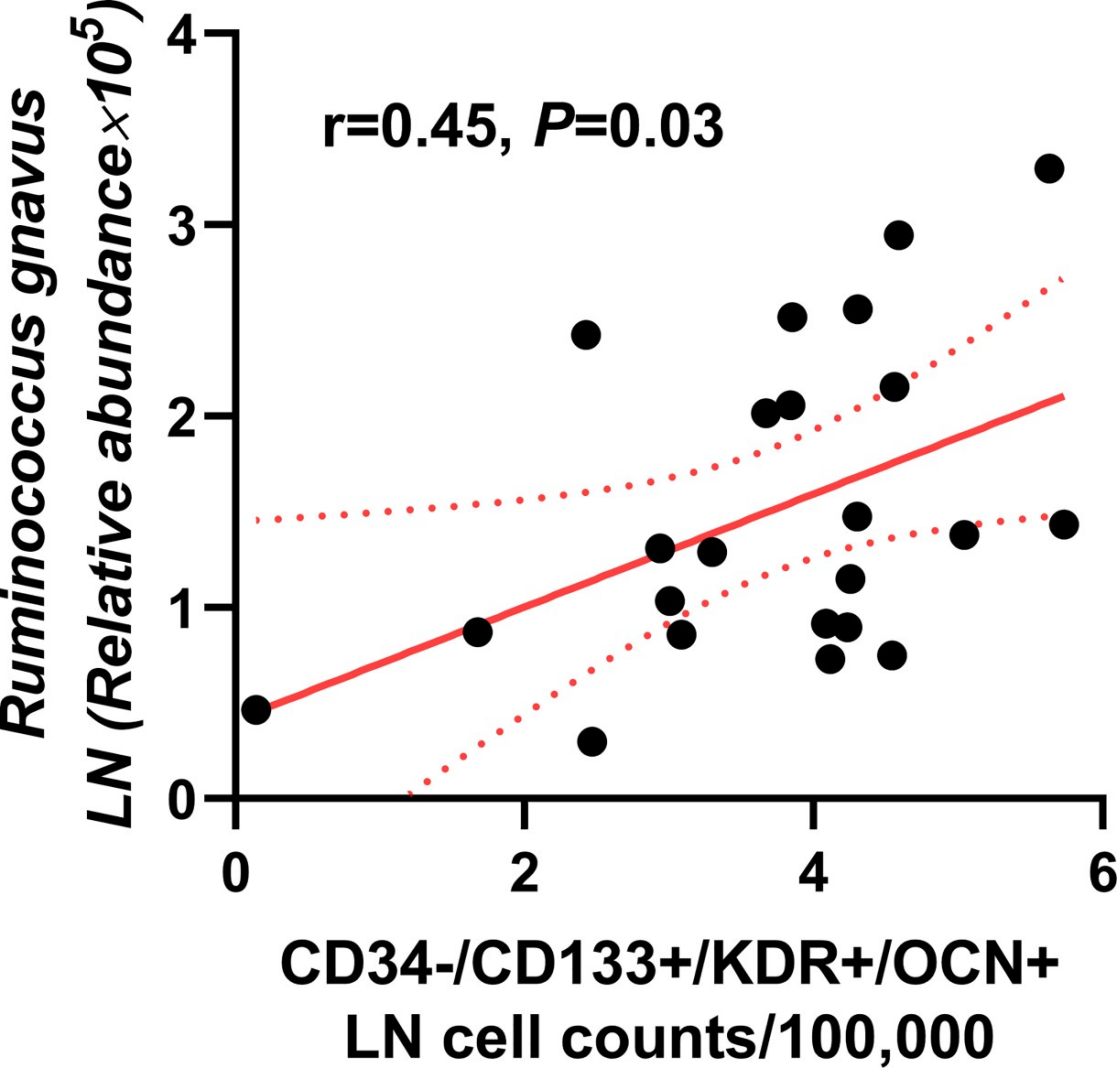
Correlation between the relative abundance of *Ruminococcus gnavus* and OCN-expressing immature EPCs. There was a moderate positive correlation between relative abundance of *Ruminococcus gnavus* and circulating CD34-/CD133+/KDR+/OCN+ cell levels in patients with diabetes mellitus (r = 0.45, *P* = 0.03).

There was a weak, but positive correlation between relative abundance of *Ruminococcus gnavus* and immature OCN-expressing EPCs (r = 0.15, *P* = 0.047) (**S3 Fig**) and it became more prominent in patients with diabetes mellitus (r = 0.45, *P* = 0.03) (**Fig 4**). Given that age is an important determinant of microbiome composition, we tested this correlation in limited age range between 30 and 69 for sensitivity analysis, yielding stronger correlation in 16 diabetic patients (r = 0.54, *P* = 0.03). Diabetic patients had higher levels of immature OCN-expressing EPCs than non-diabetic patients (4.08 [3.00, 4.54] vs. 3.27 [2.09, 4.02] LN cell counts/100,000 counts, *P* = 0.03) (**Fig 3E**). There was no significant correlation between immature OCN-expressing EPCs and relative abundance of *Lachnospiraceae NK4B4* (r = 0.05, *P* = 0.54) or *Ruminococcus Gauvreauii* (r = 0.003, *P* = 0.97), both of which were likely to be protective against CAD in our previous report [14].

## Discussion

In the present study, we demonstrated that patients with CAD had a higher prevalence of dysbiosis characterized by lower microbial richness and higher levels of TMAO than those without CAD. The circulating levels of immature but not mature OCN-expressing EPCs were significantly higher in patients with either CAD or dysbiosis than those without them. The relative abundance of the inflammatory disease-related microbe, *Ruminococcus gnavus*, was positively correlated with immature OCN-expressing EPCs, especially in patients with diabetes mellitus. The compositional alteration of the gut microbiome may affect the levels of osteogenic OCN-expressing EPCs and TMAO. The current study may further support the link between the gut microbiome and coronary atherosclerosis and vascular calcification in humans.

### OCN-expressing EPCs and CAD

We demonstrated in this study that levels of immature OCN-expressing EPCs were higher in patients with CAD than those without CAD. We previously reported that the number of mature OCN-expressing EPCs were elevated in patients with significant CAD (>50% stenosis), and also in patients with endothelial dysfunction and non-obstructive CAD than normal controls [5]. In this study, 69 (61%) out of 114 patients without CAD were patients with peripheral endothelial dysfunction (RH-PAT ≤2.0). The remaining 45 (39%) patients were not confirmed to have endothelial dysfunction by non-invasive or invasive testing; however, given that these patients were referred for clinically indicated coronary angiography because of dyspnea and/or chest pain, some of them might have endothelial dysfunction. Therefore, our current data showing no difference in the levels of mature OCN-expressing EPCs between patients with and without CAD was still consistent with our previous observation reporting no difference in mature OCN-expressing EPCs between patients with significant CAD and patients with endothelial dysfunction and no-obstructive CAD [5]. It extends our observations showing that immature OCN-expressing EPCs were higher in CAD patients than patients with cardiovascular risk factors and no-obstructive CAD [6]. More interestingly, immature OCN-expressing EPCs were even higher in patients with a history of unstable CAD than those with a stable CAD history, suggesting the aggressive contribution of this particular subset of EPCs to atherogenesis [6]. In contrast, we also reported the higher retention of mature OCN-expressing EPCs within the coronary circulation in patients with coronary endothelial dysfunction than those without coronary endothelial dysfunction [4]. Therefore, mature and immature OCN-expressing EPCs might possibly be involved in different processes leading to vascular dysfunction and calcification; the former might be involved in the vascular injury or abnormal endothelial repair, and the latter in atherogenesis.

### Dysbiosis and immature EPCs

Our current data showed that gut dysbiosis was associated with increased levels of immature OCN-expressing EPCs. We previously reported a positive relationship between OCN-expressing EPCs and vascular inflammation marker, ICAM [6]. Because TMAO increased ICAM expression in cultured endothelium, we tested our hypothesis that TMAO might be associated with the mobilization of OCN-expressing EPCs in the peripheral circulation [33]. However, TMAO levels were not associated with levels of OCN-expressing EPCs. Another potential link between dysbiosis, EPCs, and CAD is inflammation. Indeed, we found a positive correlation between the relative abundance of *Ruminococcus gnavus* and OCN-expressing immature EPCs, which was prominent in patients with diabetes mellitus. We have previously reported that the relative abundance of *Ruminococcus gnavus* increased in patients with CAD than those without CAD in the same population, possibly through production of an inflammatory polysaccharide with a resultant increase in tumor necrosis factor-α (TNF-α) [14,34]. In addition, certain clades of *Ruminococcus gnavus* have been associated with Crohn’s disease, whose inflammation is driven by TNF-α [34]. In vitro, TNF-α stimulation increased EPCs adhesion to the endothelium [35]. Mature and immature EPCs showed different responses to TNF-α stimulation; immature EPCs produced significantly higher levels of myeloperoxidase and tissue factor, and tended to produce more RANTES (regulated upon activation normal T cell expressed and secreted), a proinflammatory chemokine inducing leukocyte migration and plaque formation, than mature EPCs [36,37]. Interestingly, we observed a borderline significant association between dysbiosis and increased levels of immature EPCs without OCN expression. One possible hypothesis is that dysbiosis might promote mobilization of immature EPCs into the peripheral circulation, and microbiome-driven TNF-α and TMAO stimulation might in turn augment the inflammatory response, thus increasing their pathogenic profile. The reason for the stronger correlation between *Ruminococcus gnavus* and OCN-expressing immature EPCs in diabetic than non-diabetic patients was unclear; however, a recent study indicated that strain-specific differences in fucosidases in *Ruminococcus gnavus* might be contributory to the pathogenesis of diabetes mellitus, suggesting that the potential difference in the inflammatory process is caused by the microbial difference between diabetic and non-diabetic patients [38]. Interestingly *Rumminoccus gnavus* which added with *Clostridium symbiosum* protected underfed mice from malnutrition induced by fecal transplant from malnourished infants in Malawi, suggesting the metabolic effects of the microbe [39]; this beneficial effects observed in malnourished mice could potentially be deleterious in a well fed western population.

EPCs carrying the osteoblastic marker, OCN, indicate their osteogenic property and may mediate vascular calcification. We have shown previously that mononuclear cells from the patients with the highest levels of OCN-expressing cells were able to form mineralized deposits in the presence of calcium in vitro [5]. Additionally, we reported the positive association between OCN-expressing EPCs and the progression of aortic stenosis, and detected these cells in the calcified aortic valves [7,8]. Gut dysbiosis is a putative link between bone demineralization (osteoporosis) and vascular mineralization (calcification), known as bone-vascular axis, in the elderly, postmenopausal women, and patients with chronic kidney disease [40–43]. TNF-α is also an inhibitor of bone formation [44]. It could be postulated that increased levels of OCN-expressing cells accompanied by dysbiosis-induced inflammation might mediate this bone-vascular axis, requiring future investigation. A recent study also suggested that hyperglycemic condition might promote secretion of proinflammatory and osteogenic factors from macrophages, which might be associated with increased levels of OCN-expressing EPCs in diabetic patients [45].

### Limitations

This study has several limitations. First, the cross-sectional design makes it challenging to derive causal associations, and the results should be considered as hypothesis-generating. The study participants were selected from the patients referred for clinically indicated coronary angiography or patients with peripheral endothelial dysfunction. Selection bias cannot be avoided, thus potentially affecting the generalizability of our current observation. Though our analyses were performed based on the categorization by CAD, dysbiosis, and TMAO levels, the lack of healthy controls limits our ability to assess the normal range of OCN-expressing EPCs and normal variations of gut microbiome composition. Second, the difference in baseline characteristics between patients with and without CAD or dysbiosis might have affected the observed variation in the levels of immature OCN-expressing EPCs, since there was a paucity of data on how these potential confounders affect OCN-expressing EPCs. Age and sex are the crucial determinants of the gut microbiome composition [46,47]. In contrast, dysbiosis has also been linked to other comorbidities such as hypertension, obesity, and diabetes mellitus, leading to CAD [48–50]. Potential confounding effects were not eliminated in this study, requiring future studies to verify the current observations in other populations. Nevertheless, we have reported that gut dysbiosis was associated with CAD even after taking these confounders into account by comparing gut microbial composition between CAD patients and age-, sex-, race-, and BMI-matched non-CAD patients [14]. We also reported that increased circulating levels of immature OCN-expressing EPCs were independently associated with unstable CAD after adjusting for age, sex, and other cardiovascular risk factors [6]. Third, given that TMAO was not associated with OCN-expressing EPCs in this study, inflammation may be another potential link between dysbiosis and OCN-expressing EPCs. The lack of inflammatory markers limited our ability to mechanistically support above mentioned hypothesis, requiring future investigation.

## Conclusions

Our findings demonstrate the association between the composition of the gut microbiome and CAD may, in part, be mediated by mobilization of immature OCN-expressing EPCs. This alteration in circulating levels of OCN-expressing EPCs, which may lead to vascular calcification, may potentially be mediated by gut-microbiome-derived inflammation but not by TMAO. Future studies to explore the mechanistic role of *Ruminococcus gnavus* on atherogenesis merits further investigation.

## Supporting information

S1 Fig Circulating levels of CD34+/CD133-/KDR+/OCN+ cells were not different among 4 groups (non-CAD without dysbiosis 1.94 [1.01, 2.67] vs non-CAD with dysbiosis 2.18 [1.19, 2.92] vs CAD without dysbiosis 1.59 [0.93, 2.61] vs CAD with dysbiosis 1.73 [0.69, 2.52] LN cell counts/100,000 counts, Kruskal-Wallis test *P* = 0.55). (DOCX)

S2 Fig Circulating levels of CD34-/CD133+/KDR+/OCN+ cells were higher in CAD patients with dysbiosis than non-CAD patients without dysbiosis (non-CAD without dysbiosis 2.97 [1.68, 3.84] vs non-CAD with dysbiosis 3.30 [2.42, 3.97] vs CAD without dysbiosis 3.05 [2.13, 4.01] vs CAD with dysbiosis 3.70 [2.31, 4.36] LN cell counts/100,000 counts, Kruskal-Wallis test *P* = 0.10; non-CAD without dysbiosis vs CAD with dysbiosis, post hoc using Dunn method *P* = 0.01). (DOCX)

S3 Fig There was a weak, but positive correlation between relative abundance of *Ruminococcus gnavus* (LN [relative abundance×10^5^]) and circulating CD34-/CD133-/KDR+/OCN+ cell levels (r = 0.15, *P* = 0.047). (DOCX)

## Abbreviations

CAD: Coronary artery disease
EPC: Endothelial progenitor cell
LN: Log-transformed
MACE: Major adverse cardiovascular event
OCN: Osteocalcin
OUT: Operational taxonomy unit
TMAO: Trimethylamine N-oxide
