# Supplementary material for: Compositional change of gut microbiome and osteocalcin expressing endothelial progenitor cells in patients with coronary artery disease
Source: PLoS One. 2021 Mar 25;16(3):e0249187. doi: 10.1371/journal.pone.0249187 (PMC7993831; doi:10.1371/journal.pone.0249187)
Supplement: S1 Fig — (DOCX) [file pone.0249187.s001.docx]

**S1 Fig. Distribution of OCN-expressing mature EPC levels in CAD vs non-CAD patients with or without dysbiosis**

**
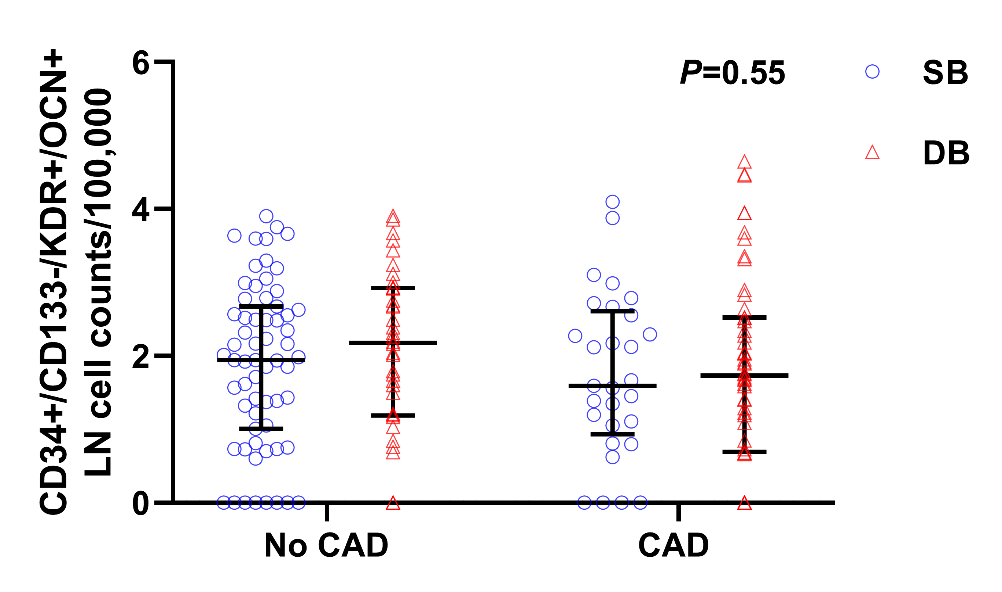
**

Circulating levels of CD34+/CD133-/KDR+/OCN+ cells were not different among 4 groups (non-CAD without dysbiosis 1.94 [1.01, 2.67] vs non-CAD with dysbiosis 2.18 [1.19, 2.92] vs CAD without dysbiosis 1.59 [0.93, 2.61] vs CAD with dysbiosis 1.73 [0.69, 2.52] LN cell counts/100,000 counts, Kruskal-Wallis test *P*=0.55).
