# Supplementary material for: Compositional change of gut microbiome and osteocalcin expressing endothelial progenitor cells in patients with coronary artery disease
Source: PLoS One. 2021 Mar 25;16(3):e0249187. doi: 10.1371/journal.pone.0249187 (PMC7993831; doi:10.1371/journal.pone.0249187)
Supplement: S2 Fig — (DOCX) [file pone.0249187.s002.docx]

**S2 Fig. Distribution of OCN-expressing immature EPC levels in CAD vs non-CAD patients with or without dysbiosis**

**
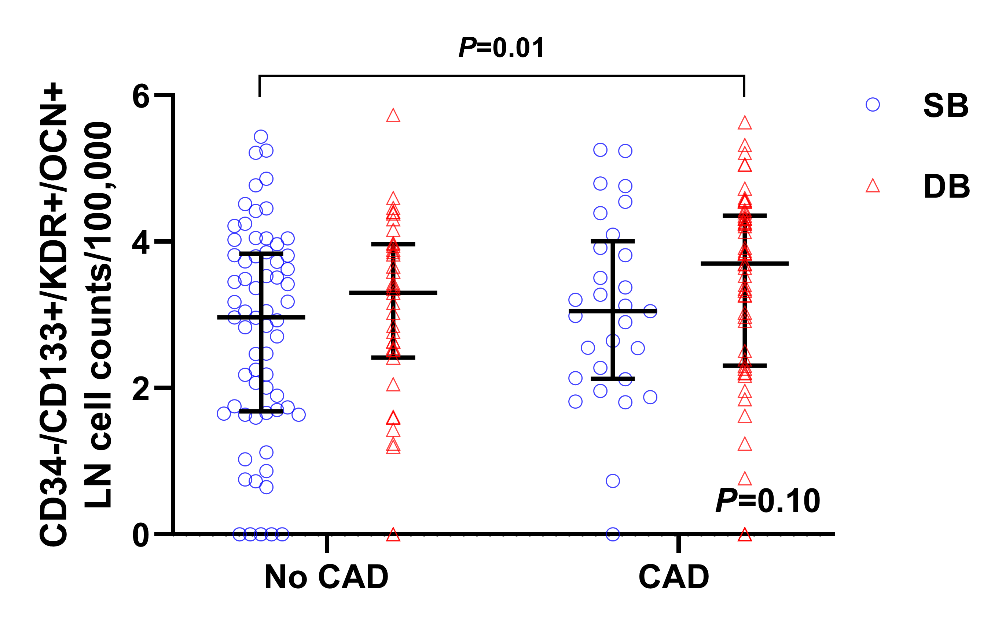
**

Circulating levels of CD34-/CD133+/KDR+/OCN+ cells were higher in CAD patients with dysbiosis than non-CAD patients without dysbiosis (non-CAD without dysbiosis 2.97 [1.68, 3.84] vs non-CAD with dysbiosis 3.30 [2.42, 3.97] vs CAD without dysbiosis 3.05 [2.13, 4.01] vs CAD with dysbiosis 3.70 [2.31, 4.36] LN cell counts/100,000 counts, Kruskal-Wallis test *P*=0.10; non-CAD without dysbiosis vs CAD with dysbiosis, post hoc using Dunn method *P*=0.01).
