# Supplementary material for: Compositional change of gut microbiome and osteocalcin expressing endothelial progenitor cells in patients with coronary artery disease
Source: PLoS One. 2021 Mar 25;16(3):e0249187. doi: 10.1371/journal.pone.0249187 (PMC7993831; doi:10.1371/journal.pone.0249187)
Supplement: S3 Fig — (DOCX) [file pone.0249187.s003.docx]

**S3 Fig. Correlation between the relative abundance of *Ruminococcus gnavus* and OCN-expressing immature EPCs**


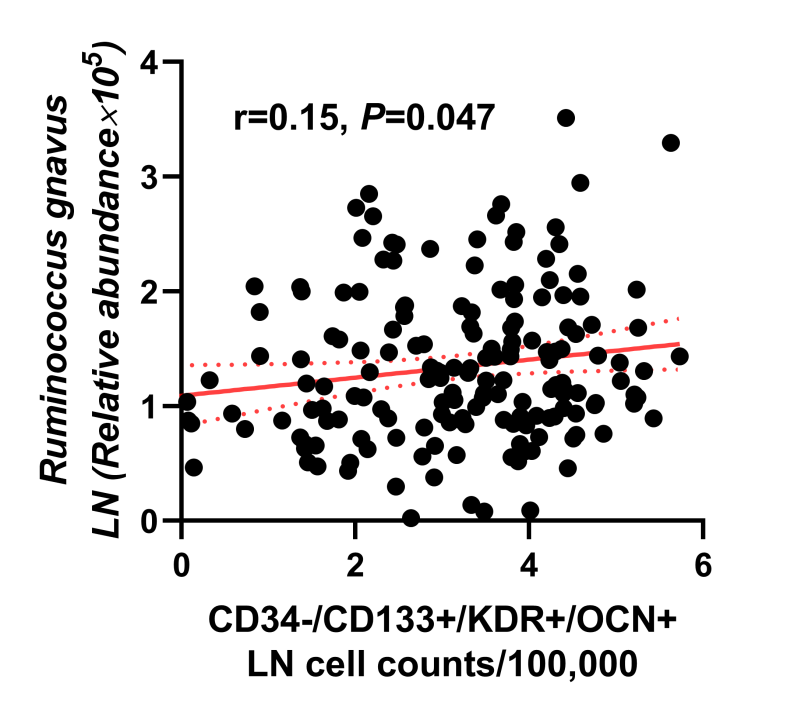


There was a weak, but positive correlation between relative abundance of *Ruminococcus gnavus* (LN [relative abundance×10^5^]) and circulating CD34-/CD133-/KDR+/OCN+ cell levels (r=0.15, *P*=0.047).
